# PIK3CA Gene Mutations in Solid Malignancies: Association with Clinicopathological Parameters and Prognosis

**DOI:** 10.3390/cancers12010093

**Published:** 2019-12-30

**Authors:** Ali Alqahtani, Hazem S. K. Ayesh, Hafez Halawani

**Affiliations:** 1 Department of Internal Medicine, University of Toledo College of Medicine and Life Sciences, Toledo, OH 43614, USA; Hazem.Ayesh@utoledo.edu; 2 Department of Oncology, Cabrini Cancer Center, Alexandria, LA 71301, USA; Halhalawani@gmail.com

**Keywords:** solid malignancy, *PIK3CA*, mutation, overall survival, PI3K/AKT/mTOR pathway

## Abstract

Phosphoinositide kinases (PIKs) are a group of lipid kinases that are important upstream activators of various significant signaling pathways. Hyperactivation of the PI3K/AKT/mTOR pathways—either via mutations or genomic amplification—confers key oncogenic activity, essential for the development and progression of several solid tumors. Alterations in the *PIK3CA* gene are associated with poor prognosis of solid malignancies. Although the literature reports contradictory prognostic values of *PIK3CA* in aggressive cancers, most of the available data highlight the important role of *PIK3CA* mutation in mediating tumorigenesis via increased signaling of the PI3K/AKT/mTOR survival pathway. Several inhibitors of PI3K/AKT/mTOR pathways are investigated as potential therapeutic options in solid malignancies. This article reviews the role of *PIK3CA* mutations and inhibitors of PI3K/AKT/mTOR pathways in major cancer types and examines its association with clinicopathological parameters and prognosis.

## 1. Introduction

Phosphoinositide kinases (PIKs) are a group of lipid kinases that act as signal transducers in various signaling pathways. They mediate signaling by phosphorylating the inositol ring of phosphoinositides.

PIKs are classified into three major families depending on the site of phosphorylation on the carbohydrate: phosphoinositide 3-kinases (PI3Ks), phosphoinositide 4-kinases (PIP4Ks), and phosphoinositide 5-kinases (PIP5Ks). PI3Ks are heterodimeric enzymes composed of catalytic and regulatory subunits. They can be categorized into classes I, II, or III, depending on the substrate specificity and their subunit structure and regulation.

**Figure 1:**
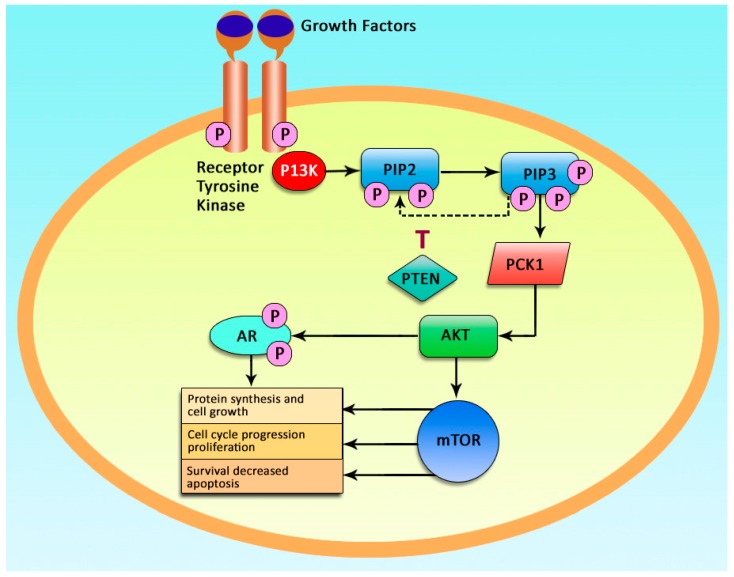
The phosphatidylinositol 3-kinase (PI3K)/AKT/mTOR pathway. Main signaling pathway initiated by growth factors that activate tyrosine kinase to initiate a series of downstream reactions that trigger P13K to generate second messenger (PIP3). PIP3 activates downstream signaling cascade important in cell proliferation, migration, transport, and survival.

PI3K is activated by a growth factor bound receptor tyrosine kinase (RTK), and once activated, it phosphorylates other signaling molecules, in a substrate specific manner, resulting in downstream conduction of chemical signals. Specifically, the activated PI3K generates the second messenger, phosphatidylinositol 3,4,5-trisphosphate (PIP3), by phosphorylating phosphatidylinositol 4,5 bisphosphate (PIP2). The activation of PI3K and subsequent production of PIP3 mediates various downstream pathways involved in several cellular functions including the pathways of tumor development and progression. PI3K signaling plays pivotal roles in cellular processes including cell proliferation, migration, transport within the cells and, most importantly, cell survival (Figure 1).

The phosphatidylinositol-4,5-bisphosphate 3-kinase catalytic subunit alpha (*PIK3CA*) gene encodes the p110α protein, the catalytic subunit of PI3K. The present review discusses the association of mutations in the *PIK3CA* p110a catalytic subunit of PI3K due to the increasing reports of the altered protein product of this gene being involved in several human cancer types. *PIK3CA* is located on chromosome 3q26.3 with a length of 34 kb. It has 20 exons that code for a protein of 124 kDa consisting of 1068 amino acids. Gene insertions, deletions, and somatic missense mutations in this gene have been reported in many human cancer types, like colon, breast, brain, liver, stomach, and lung cancers. Somatic mutations in *PIK3CA* were proposed to increase its kinase activity, resulting in cellular transformation [1].

In the year 1991, Graziani et al. were the first to show the association of PI3Ks, especially its subunit p110α, with cancer. They also showed that the kinase activity of PI3K was associated with viral oncoproteins [2]. This observation was further supported by reports of avian and murine retroviruses encoding oncogenic derivatives of the cellular *PIK3CA* and *AKT* genes, respectively [3,4].

Further investigations showed that phosphatase and tensin homolog (PTEN) dephosphorylates the 3-position on inositol head groups and, thereby, reverses the reaction catalyzed by PI3Ks. *PTEN* was observed to be a tumor suppressor gene that is found mutated in the common human tumors [5,6]. In these tumors, the *PTEN* mutation results in the constitutive activation of the PI3K pathway.

Several other studies reported the amplification of genomic regions containing *AKT, PDPK1,* or *PIK3CA* genes [7,8] in various cancer types. This implied that PI3K acted as an oncogene. Mutations in the regulatory subunit of PI3K (p85) have been reported in ovarian and colon cancers [9]. A recent study demonstrated 13% mutational frequency of *PIK3CA* in solid tumors [10]. These observations substantiated the involvement of PI3K signaling in various cancer types. The present review article discussed the role of *PIK3CA* mutations in various types of solid malignancies in terms of prevalence, potential correlation with clinicopathological parameters, and role in PI3K-targeted inhibition.

### 1.1. PIK3CA Mutations in Breast Cancer

Missense mutations in *PIK3CA* are commonly found in several types of breast cancers. The main hot spots of oncogenic mutations were exon 9 and 20, which code for kinase and helical domains of the enzyme and result in overactivation of this protein [11].

The *PIK3CA* mutations in breast cancer were initially reported by Samuels et al. [12]. In their study, only one out of 12 patients had mutation in *PIK3CA* [12]. This report instigated other research groups to comprehensively carry mutational analysis of *PIK3CA* in breast cancers [13,14]. In a very short span of time, several mutations in *PIK3CA* were discovered, making it the most frequently mutated oncogene in breast cancer. It is now believed that mutations of *PIK3CA* are found in 20–30% of all human breast cancers [13,14].

Several studies have evaluated the correlation of *PIK3CA* mutations with clinicopathological parameters such as estrogen receptor (ER)/progesterone receptor (PR) positivity, the presence of lymph node metastases, and response to therapy in breast cancers (Table 1).

Saal et al. were the first to report a definite clinicopathological correlate of *PIK3CA* mutations in breast cancer [14]. They reported that *PIK3CA* mutations were frequently seen in tumors with normally expressed *PTEN*, ER, PR, and ERBB2 genes, as well as in tumors with nodal involvement. Studies demonstrated that mutations in *PIK3CA* were more common in hormone receptor-positive and HER2-positive breast cancers [25]. In a recent study by Wu et al., it was shown that *PIK3CA* mutations were positively associated with ER-positive, PR-positive, and low Ki67 labeling index, and negatively correlated with the triple-negative breast cancer subtype [26]. *PIK3CA* mutations were not associated with age at diagnosis, tumor stage, lymph node status, tumor size, or HER2 status [26].

Various contradictory studies exist regarding the effect of *PIK3CA* mutation status on disease prognosis; *PIK3CA* mutations were reported to be correlated with poor survival rates [28,29].

Barbareschi et al. reported different effects based on mutation loci. They reported that those in exon 9 are associated with poor prognosis, while those occurring in exon 20 are associated with better prognosis [30].

Deng et al. demonstrated that *PIK3CA* mutation significantly reduced disease-free survival (DFS) compared to wild-type (WT) *PIK3CA* in patients with ER-positive tumors [31]. Subsequent studies reported that *PIK3CA* mutations were highly associated with the morphology, race, ER status, PR status, and HER2 status in breast cancer [27]. Seo et al. substantiated this observation reporting similar findings [37]. *PIK3CA* mutations were predicted to be risk factors for shorter progression-free survival (PFS) [32]. Recently, co-mutation of *TP53* and *PIK3CA* was found to be associated with poor survival in residual disease after neoadjuvant chemotherapy in breast cancer [33]. Contrary to these findings, a few reports also suggested that *PIK3CA* mutations are associated with better survival [34,35,36]. In addition, studies have reported exon 9 mutations are independently associated with early recurrence and death, whereas exon 20 *PIK3CA* mutations are associated with optimal prognosis [29].

A contradictory report highlighted that there is no association of *PIK3CA* mutation status with a prognosis of breast cancer [26].

Another important clinicopathological correlate of *PIK3CA* mutation is that they are more frequently found in lobular breast cancers as compared to ductal breast cancers [42]. Barbareschi et al. reported that this observation was specific for patients with exon 9 mutations [30].

*PIK3CA* mutations have also been correlated with response to therapy in breast cancer. Berns et al. reported that mutations in *PIK3CA* make breast cancers resistant to antibody-based therapeutic trastuzumab [38]. Eichhorn et al. have suggested that over activation of *PIK3CA* due to oncogenic mutations rendered breast cancer cells refractive to the anti-HER2 agent Lapatinib [40]. *PIK3CA* mutations were shown to reduce sensitivity to neoadjuvant chemotherapy [37,41].

Contrary to these findings, Liedtke et al. found no association between *PIK3CA* mutations and a response to anthracyline and paclitaxel-based chemotherapy [43]. Activation of the PI3K/AKT/mTOR signaling pathway contributed to the resistance to endocrine therapy in breast cancers [44]. Mutations in the *PIK3CA* have been reported to be associated with resistance to several antitumor agents such as paclitaxel, tamoxifen, and trastuzumab [45]. It has been shown that PI3K and ER pathways have a synergistic effect on tumor progression [46]. Recently, it was shown that Everolimus treatment along with chemotherapy suppressed *PIK3CA*, *ESR1*, and *GATA3* gene mutation [47].

### 1.2. Prognostic Importance of PIK3CA Genetic Mutations in Colorectal Cancer

The initial study on the involvement of *PIK3CA* mutation in colorectal cancer was reported by Samuels et al., who showed that whole-genome sequencing of colon tumors revealed a *PIK3CA* mutation frequency of 32% [12]. A recent study reported *PIK3CA* mutation frequency of 14% in Belgian colorectal cancer patients. Another recent study on Chinese colon cancer patients reported a mutation frequency of 18.94%, and these mutations were more prevalent in the right-side colorectal cancer [48]. Another study also supported this finding but showed no correlation of *PIK3CA* gene mutations with clinical parameters such as gender, age, cancer stage, or differentiation [49]. *PIK3CA* mutations were reported to be more prevalent in the “protruded-type” of colon cancer as compared to the “flat-type” colon cancers [50]. Family history or inherited predisposition did not have any effect on the frequency of *PIK3CA* gene mutations in colorectal cancer. However, the patients with inherited predisposition had mutations in the kinase domain, whereas the sporadic cases had mutations in the helical domain of the gene [51]. The mutational frequency of *PIK3CA* in colon cancers shows gender bias with more frequency in the females as compared to the males [15]. However, a recent study did not find any significant difference between male and female colon cancer patients with respect to frequency of *PIK3CA* mutations [16].

Several studies attempted to establish a correlation between *PIK3CA* gene mutations and clinicopathological parameters like survival and response to therapy. All the cases with *PIK3CA* mutations in poorly differentiated clusters had nodal metastases, high pathological TNM stage, and lymphatic invasion [52]. A recent study highlighted that *PIK3CA* mutation is associated with decreased risk of peritoneal metastases in metastatic colorectal cancer [24]. Similar to breast cancer, the effect of *PIK3CA* mutational status on prognostic outcome remains controversial. In many studies, *PIK3CA* gene mutations have been correlated with poor prognosis [22,23,53,54].

Similarly, *PIK3CA* amplifications were associated with the occurrence of diffuse-type and poorly differentiated gastric cancers and peritoneal recurrence as compared to those without *PIK3CA* amplifications. It was also demonstrated that *PIK3CA* mutations conferred resistance to colon cancer cells against anti-EGFR antibodies [17,18].

However, other studies failed to establish an association between the *PIK3CA* amplifications or mutations, and patient outcomes such as survival [19,20,21,55]. Furthermore, another study even reported *PIK3CA* mutation to be associated with good prognosis in patients with microsatellite stability (MSS) stage I-III colon cancer with a significantly increased five-year relapse-free interval in patients with *PIK3CA*-mutated MSS tumors vs. those with PIK3CA WT MSS tumors [56].

Interestingly, recent clinical trials have strongly highlighted that low-dose aspirin (100 mg/day) can act as therapy in colorectal cancer patients positive for *PIK3CA* mutations and who have undergone surgical resection in terms of reducing the risk of recurrence [57,58].

### 1.3. Prognostic Role of PIK3CA Gene Mutation in Lung Cancer 

*PIK3CA* gene amplifications have been reported in lung cancer [59,60]. The frequency of *PIK3CA* gene mutation in lung cancer varies significantly. Samuels et al. reported a low frequency of *PIK3CA* mutations (4%) in lung cancer [12,61,62], with higher frequency seen in squamous cell carcinoma (7%) as compared to adenocarcinoma (2%). Another study involving more than 700 lung cancer samples reported the *PIK3CA* mutation frequency to be ca. 2% [63]. Kawano et al., for the first time, reported the amplification of mutant *PIK3CA* alleles in cancer cells [64]. PI3K pathway alterations have been identified in over 50% of lung squamous cell carcinoma cases [65]. However, a recent study in young lung adenocarcinoma patients demonstrated the absence of *PIK3CA* gene mutations [66]. A mutation frequency of 5.36% has been reported in non-small cell lung cancer [67]. The frequency of *PIK3CA* gene mutations was higher in metastatic lung adenocarcinoma than in primary tumors [68].

*PIK3CA* mutation was significantly associated with higher risk of lung failure in patients undergoing lung stereotactic body radiation therapy [69].

*PIK3CA* mutations were reported to be associated with invasive growth, vacuolar signs, and margin lobulation on chest CT. *PIK3CA* gene mutations were shown to be associated with metastases, poor prognosis, and shorter PFS times [70].

### 1.4. The Role of PIK3CA Gene Mutations in Thyroid Cancer

Contradictory findings are reported in the literature regarding the role of *PIK3CA* gene mutations in thyroid cancers. This may be attributed to the wide variety of different types of thyroid cancer. However, these studies indicate that *PIK3CA* mutations are prevalent in aplastic thyroid cancer and follicular thyroid cancer as compared to papillary carcinoma of the thyroid [71,72,73].

In an initial study, *PIK3CA* mutations were identified in the highest proportion in the anaplastic thyroid carcinomas (16%), followed by follicular thyroid carcinomas (8%), and papillary thyroid carcinomas (2%) [74]. Subsequent studies have shown varying prevalence of mutation in different subtypes of thyroid cancer, but *PIK3CA* mutations were the most commonly found in anaplastic cancers and the least observed in papillary. For example, Wang et al. reported the frequency of *PIK3CA* mutations to be 13% in follicular thyroid carcinomas and 1% in papillary thyroid carcinomas [75]. Abubaker et al. reported a 2% frequency of *PIK3CA* mutations in papillary thyroid carcinoma [76]. Similarly, Santarpia et al. reported a 14% frequency of *PIK3CA* mutations in anaplastic thyroid cancer [77]. This distribution of PIK3CA mutations among thyroid cancer subtypes may raise the valid possibility of it playing a role towards more aggressive cancer development, mirroring the different natural history of anaplastic (most aggressive) vs. papillary thyroid cancer (least aggressive).

However, subsequent studies reported that *PIK3CA* mutations played no role in these thyroid tumor types. These studies rather highlighted the involvement of *PIK3CA* gene amplification in thyroid cancer development [78,79]. Another study reported that there is no involvement of *PIK3CA* mutations in pediatric thyroid cancer [80].

*PIK3CA* mutations have been reported to co-occur with other mutations and aberrations of relevance: for example, Xing et al. reported a protective effect of SNP rs17849071 of *PIK3CA* gene in follicular thyroid cancer [81]. BRAF and *PIK3CA* mutations cooperatively promoted anaplastic thyroid cancer [82]. Additionally, in cooperation with *KRAS* mutations, *PIK3CA* mutations were reported to be associated with metastasis in thyroid cancer. These, however, did not correlate with any of the clinicopathological parameters or prognostic parameters studied, with no effect on PFS.

Studies conducted to elucidate the role of *PIK3CA* mutations in the clinicopathological parameters and prognosis point that these mutations have minimal association with the prognosis of thyroid cancers [83]. However, the presence of *PIK3CA* mutation, along with other activating mutations, resulted in increased rates of mortality and aggressive metastasis [84].

### 1.5. PIK3CA Gene Mutations Frequency in Head and Neck Squamous Cell Cancer (HNSCC) 

*PIK3CA* gene mutations are frequently observed in head and neck carcinoma. An initial study reported *PIK3CA* mutational frequency of 11% in squamous cell carcinoma in pharyngeal cancer samples [85]. The same group further reported a higher frequency (21%) of *PIK3CA* mutation in tumors of mixed origin [85]. The mutational frequency of *PIK3CA* varied between types of HNSCC: oral squamous cell carcinomas (OSCC: 21% in cell lines and 17% in of primary tumors) [86]; nasopharyngeal carcinomas (10%; [87]); and head and neck cancer cell lines (30%; [88]). *PIK3CA* gene amplifications were also reported in oral cavity cancer cell lines [89].

HNSCC patients were reported to harbor *PIK3CA* mutations at even higher frequency, with a mutational frequency of 31% [90]. In salivary duct carcinoma (SDC), the mutation frequency of *PIK3CA* was shown to be 28% [91]. Targeted next generation sequencing revealed a mutation rate of 7.5% in OSCCs [92]. Novel mutations in *PIK3CA* were recently reported as candidate driver events in human papillomavirus (HPV)-positive OSCCs [93].

In terms of prognostication, Chou et al. reported that there was no significant relationship of *PIK3CA* mutational status with clinicopathological characteristics of the tumors [94]. However, differential response of *PIK3CA*-mutated vs. WT tumors, to PI3K-targeted therapies, remains controversial in HNSCC. While one study revealed no association between *PIK3CA* and responsiveness to PI3K-targeted drugs [94], another group reported *PIK3CA* mutations to be associated with potential benefit from matched targeted therapy in parathyroid carcinoma [95]. In a recent study evaluating the effect of nonsteroidal anti-inflammatory drugs (NSAID) on survival in head and neck cancer, patients with *PIK3CA* mutations or amplification showed prolonged disease-specific survival and overall survival with NSAID use as compared with non-NSAID users [96]. Mutations in *PIK3CA* gene were associated with improved outcomes among metastatic HPV-positive oropharyngeal cancer [97], with similar results reported in HPV-negative oropharyngeal cancer [98]. Moreover, the combination of temsirolimus, carboplatin, and paclitaxel resulted in tumor regression in head and neck squamous cell carcinomas [99].

### 1.6. Prognostic Role of PIK3CA Gene Mutations in Esophageal Cancer

Initial reports of *PIK3CA* gene mutations in esophageal cancers demonstrated that these mutations were present in 12% of squamous cell carcinomas and 6% of adenocarcinomas of the esophagus. In contrast, Akagi et al. showed no involvement of *PIK3CA* mutations in esophageal squamous cell carcinoma [100]. Recently, *PIK3CA* mutations were identified in 21.7% of chagasic megaesophagus associated with esophageal squamous cell carcinoma cases. This study also highlighted that these mutations were associated with lower survival rates [101]. In 48% of esophageal squamous cell neoplasia, *PIK3CA* was identified to be amplified [102]. It was recently shown that *PIK3CA* is the most frequently mutated gene in esophageal sarcomatoid carcinoma [103], similar to what has been described for HNSCC. A meta-analysis showed that *PIK3CA* mutation has no significant effects on overall survival and disease-free survival in esophageal squamous cell carcinoma [104]. However, patients with *PIK3CA* gene mutations in exon 9 have better disease-free survival and overall survival rates [105]. Yokota et al. showed that *PIK3CA* gene mutations are independent favorable prognostic marker in esophageal cancer patients in terms of survival [106].

### 1.7. The Importance of PIK3CA Gene Mutations in Pathogenesis of Renal Cell Cancer

The PI3K/AKT/mTOR pathway is altered in approximately 20% cases, by mutations in *PIK3CA* gene [107]. Next generation sequencing revealed the presence of *PIK3CA* gene mutations in RCC [108] but not in the clear cell subtype (cc-RCC), which is the major and most deadly RCC [109]. However, in a study that focused on cc-RCC, 20 components of the PI3K/AKT/mTOR pathway were analyzed by sequencing. In this study, *PIK3CA* amplifications or mutations were reported at 5% and were mutually exclusive with the other 19 components of the pathway.

*PIK3CA* mutations have also been reported in nephroblastomatosis or Wilms tumor [110].

### 1.8. Association of PIK3CA Genetic Mutations with Cervical, Ovarian, and Urothelial Cancer

*PIK3CA* mutations have been reported in urothelial papilloma cases [111]. *PIK3CA* gene amplification has been observed in recurrent ovarian cancer [112] and yolk sac tumors [113]. In addition, *PIK3CA* mutation was identified in 38% endometrial cancer samples [114]. Amplification of *PIK3CA* has been associated with pathogenesis of cervical cancer. Miyaki et al. reported *PIK3CA* mutations in 14% invasive cervical carcinomas, while Cui et al. reported these mutations in 8.15% of invasive cervical carcinomas [50,115]. *PIK3CA* gene mutation has also been identified in small cell carcinoma of cervix [116].

A recent study in cervical cancer showed that *PIK3CA* mutation status did not have any significant association with clinicopathological characteristics but highlighted an association with poor overall survival [117]. Somatic mutations in *PIK3CA* have been reported in low frequencies in Vulvar squamous cell carcinoma [118]. *PIK3CA* mutations were observed in 10% of bladder cancer patients [119] and are associated with cisplatin resistance and a migratory phenotype in cervical cancer cells [120].

## 2. PI3K Inhibitors

As discussed earlier, the PI3K/AKT/mTOR pathway plays a pivotal key role in solid malignancies. In addition, several PI3K inhibitors have been effectively tested in various cancer types. A recent meta-analysis involving 46 different randomized control trials highlighted that the supplementation of therapy with PI3K inhibitors significantly improved progression-free survival [121]. Hyperactivation of PI3K signaling is a hallmark of cancer, and activating mutations in this pathway are common in solid malignancies [122].

Preclinical tests have demonstrated that cancer cell lines derived from solid cancers became sensitive to hormone therapy, chemotherapy, or other targeted therapies when they are treated with PI3K or mTOR inhibitors [123]. 

The PI3K/AKT/mTOR pathway inhibitors were found to be effective in improving progression-free survival in patients with PI3K pathway mutations [121]. Table 2 summarizes different clinical trials focused on PI3K/AKT/mTOR pathway inhibitors. In the same meta-analysis it was found that these inhibitors improved overall survival in breast cancer, renal cancer, gastrointestinal cancer, head and neck squamous cell cancer, pancreatic cancer, neuroendocrine tumor and sarcomas. However, this improvement was not statistically significant and did not show any significant effect on the overall survival in other types of cancers [121].

In the year 1988, PI3K (phosphoinositide3-kinase) was first identified as a signal transducer for cell surface growth factor receptors [124]. It is one of the most commonly implicated signal pathways in a number of cancers. Although PI3K mutations were thought to be associated with oncogenesis, it was only in the year 2004, its contributory role in cancer has been established unequivocally [124]. Genetic hyperactivity of the PI3K/AKT pathway has been established as one of the most recognized underlying mechanisms for a number of cancers [124,125]. Table 3 shows a list of clinical trials that highlighted the role of mTOR and PI3K inhibitors in solid and hematological tumors therapy.

Literature search reveals that a number of studies have established the role of phosphatidylinositol-4,5-bisphosphate 3-kinase, catalytic subunit alpha (*PIK3CA*) mutations in oncogenesis.

The Cancer Genome Atlas has concluded mutations of *PIK3CA* as one of the most common genetic events associated with at least 12 different types of solid cancers [126]. Commonly-identified cancers of solid tumors associated with *PI3K3CA* are breast cancer (>30% of total cases), bladder cancer (>20% of total cases), colorectal cancer (>17% of total cancer), and squamous cell cancer of head neck region (>15% of total cases) [126,127].

In the year 2016, Millis and his colleagues explored the patterns of molecular aberration in the PI3K pathway in relation to solid tumors. From January 2013 to December 2014, 19,784 tumor samples (of more than 40 different types of cancer) sent by thousands of doctors across 60 countries were tested at a single commercial laboratory for molecular profiling to identify genetic and proteomic aberrations in the PI3K pathway [127]. They found around 38% of the patients had one or more mutations in the PI3K pathway proteins; the most common aberration (30%) was loss of PTEN (phosphatase and tensin homologue) followed by mutations in *PIK3CA* (13%). *PIK3CA* mutation was associated most commonly with endometrial (37%), breast (31%), cervical (29%), and anal cancers (27%) [128].

Again, in the year 2013, Kandoth and his colleagues explored the significance and extent of mutation in 12 types of major cancers [127]. They published the results of their analysis regarding mutations (point and small insertions or deletions) in 3281 samples of tumors (of 12 different types of tumors). According to their work *PIK3CA* was the second most common type of mutation with a frequency of >10% in majority of the cancers except ovarian serous carcinoma, renal clear cell cancer, lung adenocarcinoma and acute myeloid leukemia. Most commonly associated cancer varieties with PIK3CA mutation were endometrial cancer (uterine corpus) (52%) and breast carcinoma (33.6%).

Discovery of *PIK3CA* mutations in majority of cancers has led to new targets for treatment of those cancers. Although monotherapy with *PIK3CA* inhibitors has led to poor response [1], implementation of strategies where PI3K inhibition is tailored as per the cancer type and patients might lead to better response. Table 4 shows selected ongoing clinical trials that involved *PIK3CA* mutation as a selection population biomarker.

All these studies indicate that PI3K pathway inhibitors in solid malignancies improved progression-free survival in cancer types with PI3K mutations. However, these inhibitors are not significantly effective in improving the overall survival. Considering the side-effects of these therapies, the inhibitors should be used meticulously in treating these cancers. 

## 3. Conclusions

*PIK3CA* gene mutations are clinically important in most solid malignancies. These mutations lead to hyperactivation of the kinase activity of the PI3K pathway, resulting in deregulated cell proliferation. In addition, these mutations have implications in the effectiveness of the treatment and prognosis of cancers. However, contradictory reports exist in the literature regarding the effect of *PIK3CA* gene mutations on the prognosis of different cancers. Although different clinical trials have successfully reported the usefulness of different Pi3K inhibitors in different solid malignancies and showed a significant positive association with prognostic parameters such as progression-free survival and overall survival. However, larger prospective studies should be conducted to further elucidate the role of *PIK3CA* mutations in solid malignancies. The advent of high throughput and advanced molecular editing technologies can help evaluate the effectiveness of gene therapies and gene editing using CRISPER/Cas system in correcting genetic defects that result in, or are associated with, poor prognosis of these cancers.

## Figures and Tables

**Table 1:** Association of *PIK3CA* mutation with clino-pathological and prognostic parameters.

Cancer Type	Reference	Clinicopathological and Prognostic Parameters
Colon Cancer	[15]	Nodal metastases, high pathological TNM stage, and lymphatic invasion
	[16]	Decreased risk of peritoneal metastases
	[17,18]	Diffuse-type and poorly differentiated gastric cancers and peritoneal recurrence
		Not associated with patient outcomes such as survival
	[19,20]	Not associated with the overall survival
	[21]	Increased five-year relapse-free interval
	[22,23]	Against anti-EGFR antibodies
	[24]	Poor prognosis
Breast Cancer	[14]	Nodal involvement
	[25]	Hormone receptor positive and HER2-positive status
	[26,27]	ER-positive, PR-positive, low Ki67 labeling index and negatively correlated with triple-negative breast cancer subtype
	[28,29]	Poor survival rates
	[30]	Mutations in exon 9 are associated with poor prognosis but mutations in exon 20 are associated with better prognosis
	[31]	Reduced disease-free survival
	[32]	Risk factors for progression-free survival
	[33]	Poor survival
	[34,35,36,37]	Better survival
	[30]	Exon 9 mutations are independently associated with early recurrence and death, whereas exon 20 *PIK3CA* mutations are associated with optimal prognosis
	[38,39,40,41]	Resistant to antibody-based therapeutic therapy and chemotherapy

**Table 2:** Different PI3K/AKT/mTOR inhibitors under clinical development or approved for clinical use in SOLID tumors.

Clinical Trial/Author	Year	Tumor	Phase	Target	Protocol	Primary End-Point
Andre (BOLERO-3)	2014	Breast	III	mTORC1	Everolimus + Vinorelbine + trastuzumab vs. placebo + Vinorelbine + trastuzumab	PFS
Bachelot (GINECO)	2012	Breast	II	mTORC1	Everolimus + tamoxifen vs. tamoxifen	CBR
Baselga (BOLERO-2) **	2012	Breast	III	mTORC1	Everolimus + exemestane vs. placebo+ exemestane	PFS
Baselga (BELLE-2)	2017	Breast	III	Pan-PI3K	Buparlisib + fulvestrant vs. placebo+ fulvestrant	PFS
Baselga	2017	Breast	II	mTORC1	Ridaforolimus + dalotuzumab + exemestane vs. exemestane	PFS
Hurvitz (BOLERO-1)	2015	Breast	III	mTORC1	Everolimus+ Trastuzumab+ Paclitaxel vs. placebo+ Trastuzumab + Paclitaxel	PFS
Kim (LOTUS)	2017	Breast	II	AKT	Ipatasertib+ paclitaxel vs. placebo+ paclitaxel	PFS
Krop (FERGI)	2016	Breast	II	Pan-PI3K	Pictilisib+ fulvestrant vs. placebo+ fulvestrant	PFS
Martin (BELLE-4)	2017	Breast	III	Pan-PI3K	Buparlisib + paclitaxel vs. placebo + paclitaxel	PFS
Vuylsteke (PEGGY)	2016	Breast	II	Pan-PI3K	Pictilisib+ paclitaxel vs. placebo+ paclitaxel	PFS
Wolff (HORIZON)	2013	Breast	III	mTORC1	Temsirolimus + letrozole vs. placebo + letrozole	PFS
Yardley	2015	Breast	II	mTORC1	Everolimus +Paclitaxel+ Bevacizumab vs. Placebo + Paclitaxel+ Bevacizumab	PFS
Armstrong (ASPEN)	2016	RCC	II	mTORC1	Everolimus vs. sunitinib	PFS
Choueiri (METEOR)	2016	RCC	III	mTORC1	Everolimus vs. cabozantinib	PFS
Cirkel (ROPETAR)	2016	RCC	II	mTORC1	Everolimus + pazopanib vs. pazopanib	PFS
Dutcher#a; b	2009	RCC	III	mTORC1	Temsirolimus vs. interferon (a: cc-RCC; b: non-cc-RCC)	OS
Flaherty#a; b; c (ECOG2804)	2015	RCC	II	mTORC1	(a) Bevacizumab + temsirolimus vs. bevacizumab alone (b) Bevacizumab + temsirolimus vs. bevacizumab + sorafenib (c) Sorafenib + temsirolimus vs. bevacizumab + sorafenib	PFS
Hudes#a; b **	2007	RCC	III	mTORC1	(a) Temsirolimus vs. interferon (b) Temsirolimus + interferon vs. interferon	OS
Hutson	2013	RCC	III	mTORC1	Temsirolimus vs. sorafenib	PFS
Motzer (RECORD-1) **	2008	RCC	III	mTORC1	Everolimus vs. placebo	PFS
Motzer (RECORD-3)	2014	RCC	II	mTORC1	Everolimus vs. sunitinib	PFS
Motzer	2015	RCC	III	mTORC1	Everolimus vs. Nivolumab	OS
Negrier (TORAVA)	2011	RCC	II	mTORC1	Temsirolimus + bevacizumab vs. INF-α + bevacizumab	PFS
Rini (INTORACT)	2013	RCC	III	mTORC1	Temsirolimus+ bevacizumab vs. IFN + bevacizumab	PFS
Tannir	2015	RCC	II	mTORC1	Temsirolimus vs. sunitinib	PFS
Besse	2014	Lung	II	mTORC1	Everolimus + erlotinib vs. erlotinib	DCR
Levy	2014	Lung	II	Pan-PI3K	PX-866+ docetaxel vs. docetaxel	PFS
Papadimitrakopoulou (BATTLE-2)	2016	Lung	II	AKT	MK-2206+erlotinib vs. erlotinib	DCR
Socinski (TAX 326)	2010	Lung	II	AKT	Enzastaurin+ carboplatin vs. carboplatin	TTP
Zhu (EVOLVE-1)	2014	Lung	III	mTORC1	Everolimus vs. placebo	OS
Bendell	2011	CRC	II	PI3K/Akt/mTOR pathway	Perifosine + capecitabine vs. placebo + capecitabine	TTP
Bowles	2016	CRC	II	Pan-PI3K	PX-866 + cetuximab vs. placebo + cetuximab	PFS
Ohtsu (GRANITE-1)	2013	Gastric cancer	III	mTORC1	Everolimus vs. placebo	OS
Jimeno	2015	HNSCC	II	Pan-PI3K	PX-866 + cetuximab vs. cetuximab	PFS
Jimeno	2016	HNSCC	II	Pan-PI3K	PX-866 + docetaxel vs. docetaxel	PFS
Soulieres (BERIL-1)	2017	HNSCC	II	Pan-PI3K	Buparlisib + paclitaxel vs. placebo + paclitaxel	PFS
Rachards	2011	Pancreatic	II	AKT	Enzastaurin + gemcitabine vs gemcitabine	OS
Pavel (RADIANT-2)	2011	NET	III	mTORC1	Everolimus + octreotide LAR vs placebo+ octreotide LAR	PFS
Yao (RADIANT-3) **	2011	NET	III	mTORC1	Everolimus vs. placebo	PFS
Yao (RADIANT-4) **	2016	NET	III	mTORC1	Everolimus vs. placebo	PFS
Eroglu	2015	Sarcoma	II	mTORC1	Temsirolimus + selumetinib vs. selumetinib	PFS
Demetri	2013	Sarcoma	III	mTORC1	Redaforolimus vs. placebo	PFS
Oza	2015	Endometrial cancer	II	mTORC1	Ridaforolimusvs progestin or chemotherapy	PFS
Wick (EORTC 26082)	2016	Glioblastoma	II	mTORC1	Temsirolimus vs. temozolomide	OS
Margolin (S0438)	2012	Melanoma	II	mTORC1	Temsirolimus+ sorafenib vs. tipifarnib+ sorafenib	PFS

**: Trials leading to product FDA approval; PFS: Progression-free survival; OS: Overall survival; IFN: interferon; TTP: Time to progression; CBR: Clinical benefit rate; DCR: Disease control rate; mTORC1: Mammalian target of rapamycin complex1; RCC: Renal cell carcinoma; cc-RCC: Clear cell-RCC; NET: Neuroendocrine tumor; HNSCC: Head and neck squamous cell carcinoma; CRC: Colorectal cancer.

**Table 3:** Clinical trials that lead to mTOR and PI3K inhibitors in solid and hematological tumors.

Biomarker	Drug	Target	Population	Study Phase	Clinicaltrials.gov Registration
*PIK3CA* mutation or amplification	Sirolimus	mTROC1	Advanced-stage solid cancers	II	NCT02449564
*PIK3CA* mutation or amplification or *PTEN* loss	Copanlisib	Pan-PI3K	Advanced HNSCC	I/II	NCT02822482
*PIK3CA* mutation	Alpelisib + fulvestrant	PI3K-α	Advanced-stage HR+/HER2− breast cancer	III	NCT02437318
	Alpelisib + fulvestrant ± letrozole	PI3K-α	Advanced-stage HR+/HER2− breast cancer	II	NCT03056755
	Taselisib	PI3K-α	Advanced-stage SCC of the lung	II	NCT02154490
*PIK3CA* and/or *BRAF* mutations	ASN003	PI3K-α AND BRAF	Advanced-stage solid cancers	I	NCT02961283
*PIK3CA, AKT,* or *PTEN* mutations	MK-2206	AKT	Advanced-stage lung and thymus cancers	II	NCT01306045
	Ipatasertib + paclitazel	AKT	Advanced-stage breast cancer	III	NCT03337724
*PIK3CA* mutation or amplification	AZD5363 + paclitaxel	AKT	Advanced-stage gastric cancer	II	NCT0251956
*PIK3CA* or *AKT* mutations	Miransertib + carboplatin	AKT	Selected advanced-stage solid cancers	I	NCT02476955

HR: Hormone receptor.

**Table 4:** Selected ongoing clinical trials of PI3K/AKT/mTOR inhibitors involving *PIK3CA* mutation as a selection population biomarker.

Drug	Target(s)	Trial	Population (*n*)	Results	Toxicities (Most Common)	Ref.
Temsirolimus	mTORC1	Global ARCC (vs. INF-α vs. combination)	Untreated, mRCC (*n* = 626)	↑ OS (10.9 vs. 7.3 months; *p* = 0.008) ↑ PFS in the temsirolimus monotherapy (*p* < 0.001)	Rash, HG, HL; mild	[5]
Everolimus	mTORC1	RECORD-I (vs. placebo)	Previously treated, mRCC (*n* = 272)	↑ PFS (4.0 vs. 1.9 months; *p* < 0.0001) No significant improvement in OS or in ORR	Stomatitis, rash, fatigue, pneumonitis, diarrhea	[6]
		RADIANT-3 (vs. placebo)	Advanced pancreatic NET (*n* = 410)	↑ PFS (mPFS 11.0 vs. 4.6 months; *p* < 0.001) No clear ORR benefit	Stomatitis, rash, fatigue, pneumonitis, diarrhea	[7]
		RADIANT-4 (vs. placebo)	Other NET (*n* = 302)	↑ PFS (11.0 vs. 3.9 months; *p* < 0.00001) No clear ORR benefit	Stomatitis, rash, fatigue, pneumonitis, diarrhea	[8]
+AI		BOLERO-2 (vs. placebo + AI)	HR+/HER2− breast cancer (*n* = 724)	↑↑ ORR (9.5% vs. 0.5% *p* < 0.001) ↑ mPFS (6.9 vs. 2.8 months; *p* < 0.001)	Stomatitis, rash, fatigue, pneumonitis, diarrhea	[9]
Copanlisib	Pan-PI3K	CHRONOS-1 (vs. placebo)	r/r B-NHL, Macroglobulinemia (*n* = 142)	ORR of 59% (12% CR and 47% PR), with a mPFS of 11.2 months	HG, nausea	[12]
Idelalisib + rituximab	PI3K-δ	NCT01539512 (vs. placebo+ rituximab)	Relapse CLL (*n* = 220)	ORR (81% vs. 13%), ↑ PFS at 24 weeks (93% vs. 46%; *p* < 0.001), ↑ 1-year OS (92% vs. 80%; *p* = 0.002).	Diarrhea, rash, immune-mediated hepatitis/ pneumonis	[14]
		NCT01282424 (vs. placebo)	r/r B-NHL (FL) and SLL (*n* = 125)	ORR: 54% in FL patients and 58% in SLL (*p* < 0.001)	Diarrhea, rash, immune-mediated hepatitis, and pneumonitis	[15]

mRCC: Metastatic renal cell carcinoma; OS: Overall survival; PFS: Progression-free survival; mPFS: median PFS; ORR: Objective Response Rate; r/r: Recurrent/relapsed; NET: Neuroendocrine tumors; CLL: Chronic lymphocytic leukemia; SLL: Small lymphocytic leukemia; B-NHL: B-non Hodgkin lymphoma; HR: Hormone receptor; FL: Follicular lymphoma.
